# Child Mortality Transition in the Arabian Gulf: Wealth, Health System Reforms, and Development Goals

**DOI:** 10.3389/fpubh.2019.00402

**Published:** 2020-01-17

**Authors:** Asharaf Abdul Salam, Rshood M. Al-Khraif

**Affiliations:** Center for Population Studies, King Saud University, Riyadh, Saudi Arabia

**Keywords:** public health, social sciences, health services administration, health care economics, demography, population

## Abstract

**Background:** Child mortality is the most crucial indicator of national progress and a reflection of not only the health system performance but also the wealth (budget) utilization and goal achievements. Many developing nations have recorded progress in this dimension but those of the Arabian Gulf (Bahrain, Kuwait, Oman, Qatar, Saudi Arabia, and United Arab Emirates) show remarkable progress and achievements.

**Methods:** Using the latest update of United Nations Inter-agency Group for Child Mortality Estimation 2017, an attempt is made here to review and appraise their achievements in child mortality reduction since 1950s taking into account Under 5 mortality, infant mortality, and neonatal mortality.

**Results:** This review finds a rapid decline in child mortality in the Arabian Gulf in a short span of 50 years, which is in line with the achievement of Sustainable Development Goals.

**Conclusions:** There is a remarkable budget allocation and investment in health system building, improving the other contributing sectors like water, sanitation, hygiene, nutrition, and life style modifications apart from the usual health care interventions.

## Introduction

Child mortality decline, a universal phenomena (1) reflects the degree of progress achieved by a country in terms of social and economic development, is facilitated by a multiplicity of factors of financial, systematic, programmatic, managerial, and target oriented actions. These involve multisectoral cooperation, introduction and expansion of routine immunizations, implementation of standardized diagnosis and treatment guidelines, sophisticated decision making, prioritization of resources, treatment technologies, improvement in quality of care, coverage of health programs, improvements in health sector, and gains outside health sector (2–6). On the other hand, sectors such as nutrition, education, early child development, vaccination, HIV/AIDS, water, sanitation, and hygiene work together in an attempt to achieve carefully crafted targets ensuring the above mentioned ingredients of child mortality decline, especially in an era of pledging to reduce child mortality rate: possibly implying on health policies (4, 5, 7, 8). Additionally, observations from the Muslim majority countries have confirmed the influence and coverage of essential interventions across the continuum of care, especially of reproductive health, prenatal care, delivery and labor, and childhood vaccines not only on child mortality but also on other health outcomes (9). As of the Arab countries, there are evidences of declining premature death and disability due to communicable diseases, and nutritional complications of newborns, and mothers (10).

The Arabian Gulf countries, under the auspices of the Gulf Health Council, set an example of rapid child mortality transition despite a slow social progress but with a rapid rise in the economy—both national and per capita (11–16). This aligns with the argument that child mortality declines, despite poverty and slow socio economic progress but with programmatic implications and prioritization by national leaders, policy makers, development partners, and funders towards an improved health outcome (9). Efforts to achieve development goals of child survival create building blocks for strengthening the entire health system, which, in turn, paves way for access to quality services, affordable and respectful, at community level leading to strengthened local institutions and skilled and gender sensitive community health workers (10, 17, 18). Such developments by encouraging people, and religious communities demand a shift in service modality to primary care with proactive and continuous services (19), thereby, endorses the sustainable development goals (SDGs) of control of preventable child and maternal mortality, in turn, stresses the means to turn them around to make good health (20).

Child mortality is closely interrelated with the fertility and overall mortality levels but has been affected by abortions, miscarriages, ectopic pregnancies, and hypertensive disorders leading to stillbirth, delivery complications, birth under inadequate conditions, parasitic and communicable diseases, diarrhea, etc., causing new born deaths (8, 9). Parallel to these are the efforts in interventions in reproductive, maternal, new born, child, and adolescent health combined with contraception, infant and child feeding, and interventions for treating sick children coupled with family planning interventions, antenatal care, skilled birth attendance, and protection of newborns against tetanus and other vaccines. These combined efforts of government and other stake holders ensure provisions of human resources, funding, infrastructure equipment, and supplies of reproductive, maternal, new born, child, and adolescent health in general, especially at unstable conflict affected areas (11–16). However, addressing sustainable development goals ambitiously through understanding the gaps to improve child survival (3), helps in extending access to high quality health care, eliminating risk factors of child deaths, and bolstering socio-economic factors affecting child survival directly.

## Methodology

### Data

This study uses modeled global estimates agreed upon by the respective national authorities and are published by UN Inter-agency Group for child Mortality Estimation (UN IGME), as 2017 release, in consultation with the respective governments—Ministry of health and national statistics offices (1).

These statistical estimates resulted from a composite process-model-extrapolation-output offering (i) U5MR, the under-five mortality rate (probability of dying between birth and exactly 5 years of age per 1,000 live births), (ii) IMR, the infant mortality rate (probability of dying between birth and exactly 1 year of age per 1,000 live births), and (iii) NMR, the neonatal mortality rate (probability of dying in the first 28 days of life per 1,000 live births). Here, for the purpose of this discussion, median level estimations (Uncertainty bounds-median) pertaining to the Arabian Gulf Countries such as Bahrain, Kuwait, Qatar, Oman, Saudi Arabia, and United Arab Emirates (UAE) were considered. These statistics refers to the nation as a whole—the total population–not a sample (refer https://childmortality.org and https://data.unicef.org). The raw data of deaths of children (Under 5, infants, and neonates) and the mortality rates (U5MR, IMR, and NMR) are extracted for the six countries of Arabian Gulf for meaningful comparisons and interpretations for a trend analysis to trace the path of achievements.

### Method

Further, the health system development was reviewed for the region, with special reference to the individual countries. Based on the performance, the overall period would be divided into (a) prior to 1970; (b) 1970–2000; and (c) since 2000 corresponding to uprooting stage, expanding stage, and reform stage. Thereafter, the variables of child mortality transition appraised through stages, in terms of inputs and transformation process within the health system. This database shows data from 1961 for Bahrain; 1964 for Kuwait; 1967 for Oman; 1974 for Qatar; 1980 for Saudi Arabia; and 1967 for UAE. Considering the data availability, that pertaining to the overall GCC was calculated to understand the performance of the region. Even though, the mortality rates were available, the number of deaths for Saudi Arabia were available only from 1980; thus, the GCC regional figure starts only from that point (The relevant data are presented in the Appendix 1). For further clarity, a correlation analysis has been performed for each country and for the region-U5MR, IMR, and NMR, by taking year wise data since the earliest available till 2016.

## Results

**Figure 1:**
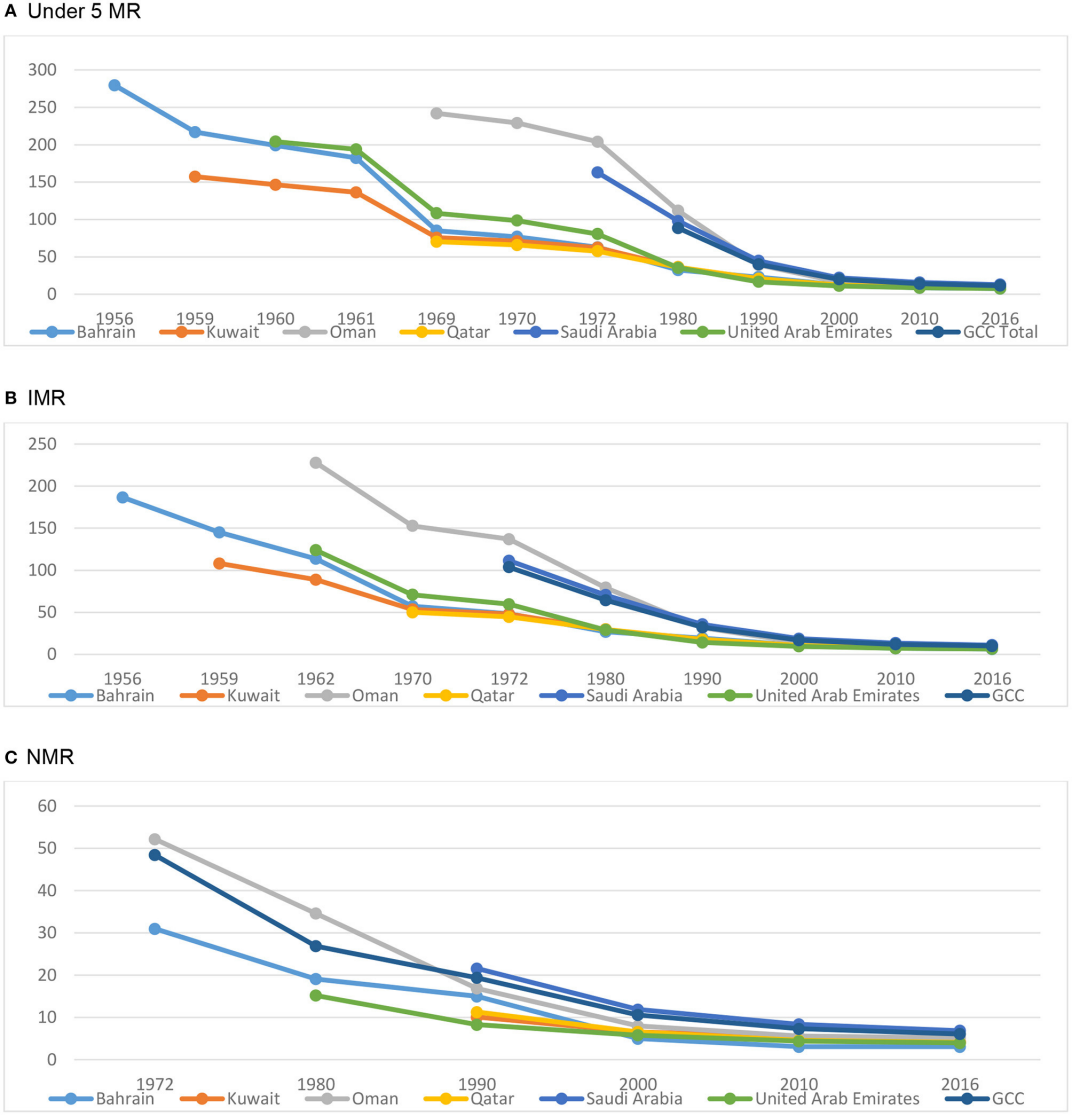
Child mortality in the Arabian Gulf since 1956. **(A)** Under 5 MR, **(B)** IMR, **(C)** NMR. Source of data: UN Inter-agency Group for child Mortality Estimation (UN IGME), as 2017 release (www.childmortality.org). Data used in plotting this graph is shown in Appendix 2.

The Figure 1A shows a concentration of U5MR, that too at a remarkably low level, below 10, although the rates diverged, noticeably, prior to 2000. However, the period till 1970 witnessed a falling U5MR, haphazardly: this could be considered as a period of unplanned change. By 1970, almost all countries reached a level close to 100, except the two large countries namely Saudi Arabia and Sultanate of Oman. Both of them took another 10 years (by 1980) to reach at par with the other four small countries (Bahrain, Kuwait, Qatar and UAE). Thereafter, till 2000, a threshold to reach a point for all the countries have been observed. As evident, by 2000, the U5MR reached a stable low level and continues as such, in all countries. Overall, this transition experience is worth learning.

On the other hand, as of 2016, there were 9,089 infant deaths in the region, of which a majority were in Saudi Arabia, obviously, the country with the largest population share. Moreover, a clear trend of declining infant deaths observed, across the region in all countries. The reductions in infant deaths is highly impressive (from 37,207 in 1980 to 9,089 in 2016) (Figure 1B). At the same time, the neonatal deaths, the most pressing area of intervention and a grave concern in the developing countries, also proves to be in transition, in the Arabian Gulf (Figure 1C).

The correlation analyses conducted have shown significance along variables: U5MR - IMR, U5MR - NMR, and IMR - NMR at *p* < 0.01 level for all countries and for the region. Such positive correlations are supportive of the community dynamics and progress (Table 1).

**Table 1:** The significance level (*p*-values) of correlation coefficients.

**Countries**	**U5MR-IMR**	**U5MR-NMR**	**IMR-NMR**
Bahrain	^**^	^**^	^**^
Kuwait	^**^	^**^	^**^
Oman	^**^	^**^	^**^
Qatar	^**^	^**^	^**^
Saudi Arabia	^**^	^**^	^**^
UAE	^**^	^**^	^**^
Total GCC	^**^	^**^	^**^

** *p < 0.01*.

## Discussion

Trends in child mortality would be assessed along the structural and contextual factors, especially state governance, conflict, and women empowerment (9). Thus, the overall scenario within the resource setting brings out the pathways that paved the way for such a rapid achievement. As has pointed out, national resources facilitates system building including routine medical records that ensures quality of care and procedural standards: this embodies the Arabian Gulf country experience in child mortality (2). Probably, with the emergence of financial resources, health budget allocations have increased to encourage program spending and target achievement: thus enabling progress in child mortality.

As these countries have formed during the 1950s after rigorous efforts of regional bifurcations, and border settings: the period till 1970s could be considered as a period of unplanned change. The fast decline till 2000 could be considered as a threshold decline, a rapid transition stage. This could probably, be due to women empowerment, and social support building to basic health care services including family planning for the overall health and nutritional care of family (9).

Probably, questions about religion in this context of Arabia deserves attention as is Islam always attributed as culprit, structural issue or one of the factors, however, it is the heterogeneity in health service coverage to adolescents and, women, that govern access to services, which in turn, improve child survival (3, 9, 17). Moreover, funding for health of new born and children serves as a key maternal and child health intervention that facilitates girl's education, status of women, and thus, women's role in decision making, particularly at local levels (3, 17). For e.g., instances of introduction and scaling up of cost effective interventions such as expansion of measles, immunization, screening and treatment of maternal syphilis and diarrhea not only reduced child mortality in many countries but also uplifted women. Probably, such interventions' sensitivity to community improve practices and promote healthy behaviors such as birth spacing, exclusive breastfeeding, and care for lactating mothers within the Islamic/Arab culture which create greater intergenerational agreement for a conducive environment to improve health outcomes, including child survival (11–17). Added to this is the healthy migrant effect, which benefits health interventions and health care improvements (21).

It is the commitment and accountability of the governance that translated resources spend into country wide child mortality indicators; witnessing timely reforms and revisions of public health policies and programs, thus paving way for technical and professional enrichment of various sectors contributing to child mortality (11–16). It is argued that absence of terrorism, greater political rights, government's effectiveness, improvements in gross national product, income per capita, higher adult literacy, greater female to male enrolment in secondary school are facilitators of decline in Under 5 mortality (9). Probably, controlling the leading causes of U5MR such as preterm births complications, pneumonia, and intra partum related events, in addition to the childhood infections and injuries have contributed to this achievement, in the Arabian Gulf countries (5).

A clear trend of IMR decline observed, a remarkable achievement in infant mortality transition, which could be attributed to the decline in fertility coupled with improvements in maternal and child health care through the health system's sensitiveness to reproductive health interventions. Probably, the reductions in NMR could have been a reflection of maternal health, labor and delivery care innovations and methodologies for averting preventable deaths through interventions in reproductive health, prenatal care, delivery, and vaccinations (7, 9). Major causes of neonatal mortality, as revealed are low birth weight and preterm births–both demands interventions, investments and health system strengthening (22). Efforts in these lines, in turn, ensure progress of preventable deaths from pneumonia, diarrhea, malaria, and measles and hospital deliveries with complete immunization (23).

These results conform to the arguments that neonatal deaths are born out of diarrhea, pneumonia, malaria, measles, meningitis, and injuries: all are higher in Muslim majority countries than others (9). But, despite being Muslim majority and also Arab, this region have managed to divert their resources effectively to health sector so as to make an impact on public health, i.e., to reduce overall mortality and more specifically, the childhood mortality. Again, the high but rapidly declining fertility would also play a role in delivery care and child rearing practices: the influencers of child mortality. Moreover, newborn deaths are averted through action plans for pneumonia, and diarrhea coupled with strategies to address adolescent health, nutrition, and sexual and reproductive health among young women, as also strategies to promote breast feeding, nutritional care, and early child development. These actions, in turn, build a platform to effectively promote community behavior, health and immunization (9).

At the outset, when everything favorable, it is the out of pocket expenditures that influences the health care utilization as well as the childhood mortality, for which government investment is the action plan (9). However, the Arabian Gulf countries pursued them with interventions to improve public health coverage. On the other hand, child mortality decline is attributed to improved levels of health determinants and GDP growth's potential that lifts multi sectoral progress (6): these, in turn, raises the importance of health through influencing perceptions and attitudes. Moreover, substantial progress in reducing disparities and inequalities in child mortality across the socio-economic groups is an accomplishment, in the Arabian Gulf. Definitely, the Arabian Gulf countries have a situation different from that of other Arab countries in terms of reduced child mortality accomplished through child health, in addition to multiple interventions targeting childhood illnesses and heightened socio-economic development (3).

Efforts in line with abortions, *in vitro* fertilizations and genetic screening techniques would also have facilitated child mortality in these Arabian Gulf countries, to an extent, along with social sector interventions such as raising age at marriage, age at first delivery, increasing female education, banning female genital mutilation, domestic violence prevention, provision of social support, and mental health services (9). Administration and execution of child survival programs shall be based at grass root level through health program planning and implementation addressing challenges of funding and targeting interventions for the needy population (3). This emphasizes the need for prioritizing the potential of women and adolescents as agents of change through greater investment across health, education, and economy aiming towards achieving 2030 agenda (17).

Added to this is the creation of geopolitical data to strengthen the precision of local monitoring of child health needs: a broad array of health sector interventions and social determinants (3, 6), in addition to these are the substantial, sustained commitments to financing better access to improved health care and targeting interventions to high burden areas. Also to build is a multisectoral collaboration offering unprecedented opportunity to build lessons from the MDGs and promote health and sustainable development (6). Perhaps, it lifts the children beyond survival but to thrive: a promise of sustainable development goals (17): accelerated investments to achieve the targets and maintain progress in line with disease control and prevention (8).

## Conclusions

Setting standards in child mortality, an arduous task, has been simplified by the targets set up as Development Goals. But to catch up with those targets, enormous efforts involving money, material and manpower are essential, which only a few countries are fortunate to devote a huge budget to accumulate equipments and technologies; develop a resourceful manpower at various levels starting with the grassroot (implementation) to the top (decision making) level for planning, organizing, directing, and controlling not only of the health system but also of the other contributing sectors like water, sanitation, nutrition, air, personal hygiene, etc. All of these requires concerted and committed efforts with accountability, which involves investments in terms of capacity building, awareness promotion, and support and assistance to meet basic needs. The fortunate Arabian Gulf region has made remarkable progress through visionary leaders in achieving a desired child mortality level through mobilizing resources, building infrastructure, hiring and grooming manpower, building systems and subsystems, and creating institutions and structures.

Achievements in child mortality in the Arabian Gulf region seems to be attractive, that too, in a short period, since 1970. This demarcate the transition into three stages—unplanned change (till 1970), threshold decline (1970–2000), and stable low level (2000–2016). These strategic achievements in child mortality have been appreciably resulted from the commitments and integrated efforts of local, national, and international levels through cooperation and initiatives. Moreover, the root causes of childhood mortality were addressed through coordinated actions from various sectors especially the water borne diseases, delivery complications, maternal health, prenatal and postnatal medical care, institutionalization of delivery, and so on. Together, this change in approaches, timely actions, investments in public programs and people oriented actions would have promoted the national health scenario and thus the child mortality.

No doubt, Arabian Gulf countries have created policies and provisions for meeting the challenges of child mortality essentially through health reforms, system creations, and thereby addressing developmental goals. This might have incurred wealth and resources in terms of health budget allocations and program planning at various levels (tiers) of health care that too on specialities like pediatric, reproductive, and maternal.

## Data Availability Statement

Publicly available datasets were analyzed in this study. This data can be found here: www.childmortality.org.

## Author Contributions

AS: conceptualization, data selection, analysis, interpretation, and writing. RA-K: advices on data selection, analysis, and discussions.

### Conflict of Interest

The authors declare that the research was conducted in the absence of any commercial or financial relationships that could be construed as a potential conflict of interest.

## References

[1] United Nations Inter-agency Group for Child Mortality Estimation (UNIGME). Levels & Trends in Child Mortality: Report 2017, Estimates Developed by the UN Inter-Agency Group for Child Mortality Estimation. New York, NY: United Nations Children's Fund (2017).

[2] King C McCollum ED. Quality of care for paediatric admissions: is a score-based approach viable? Lancet. (2018) 6:e128–9. 10.1016/S2214-109X(18)30006-8 29389528 PMC5897706

[3] Golding N Burstein R Longbottom J Browne AJ Fullman N Osgood-Zimmerman A. Mapping under 5 and neonatal mortality in Africa, 2000-15: a baseline analysis for the Sustainable Development Goals. Lancet. (2017) 390:2171–82. 10.1016/S0140-6736(17)31758-0 28958464 PMC5687451

[4] Watson SI Lilford RJ Mortality decrease according to socio-economic groups. Lancet. (2017) 389:1794–5. 10.1016/S0140-6736(17)31157-1 28495163

[5] Byass P. Child mortality is (estimated to be) falling. Lancet. (2016) 388:2965–7. 10.1016/S0140-6736(16)32169-9 27839856

[6] Bishai DM Cohen R Alfonso YN Adam T Kuruvilla S Schweitzer J. Factors contributing to maternal and child mortality reductions in 146 Low- and middle-income countries between 1990 and 2010. PLoS ONE. (2016) 11:e0144908. 10.1371/journal.pone.0144908 26783759 PMC4718632

[7] Loewenberg S Next steps for UNICEF. Lancet. (2018) 2:166 10.1016/S2352-4642(18)30032-4

[8] Liu L Oza S Hogan D Chu Y Perin J Zhu J. Global, regional, and national causes of under-5 mortality in 2000-15: an updated systematic analysis with implications for the Sustainable Development Goals. Lancet. (2016) 388:3027–35. 10.1016/S0140-6736(16)31593-8 27839855 PMC5161777

[9] Akseer N Kamali M Buckhache N Mirza M Mehta S Al-Gashm S. Status and drivers of maternal, newborn, child and adolescent health in the Islamic world: a comparative analysis. Lancet. (2018) 391:1–20. 10.1016/S0140-6736(18)30183-1 29395272

[10] Mokdad AH Jaber S Aziz MIA AlBuhairan F AlGhaithi A AlHamad NM. The state of health in the Arab world, 1990–2010: an analysis of the burden of diseases, injuries, and risk factors. Lancet. (2014) 383:309–20. 10.1016/S0140-6736(13)62189-3 24452042

[11] Regional Heatlh System Observatory Health System Profile: Bahrain. Cairo: Cairo Regional Office for the Eastern Mediterranean (2007).

[12] Regional Heatlh System Observatory Health System Profile: Kuwait. Cairo: Cairo Regional Office for the Eastern Mediterranean (2006).

[13] Regional Heatlh System Observatory Health System Profile: Qatar. Cairo: Cairo Regional Office for the Eastern Mediterranean (2006).

[14] Regional Heatlh System Observatory Health System Profile: Oman. Cairo: Cairo Regional Office for the Eastern Mediterranean (2006).

[15] Regional Heatlh System Observatory Health System Profile: Saudi Arabia. Cairo: Cairo Regional Office for the Eastern Mediterranean (2006).

[16] Regional Heatlh System Observatory Health System Profile: United Arab Emirates. Cairo: Cairo Regional Office for the Eastern Mediterranean (2006).

[17] Mohammed A. A call to action: improving women's, children's, and adolescents' health in the Muslim world. Lancet. (2018) 391:1–2. 10.1016/S0140-6736(18)30182-X 29395270

[18] Al-Yousuf M Akerele TM Al-Mazrou YY. Organization of the Saudi health system. East Mediterr Health J. (2002) 8:645–53. Available online at: http://apps.who.int/iris/handle/10665/19213 15603048

[19] Khoja T Rawaf S Qidwai W Rawaf D Nanji K Hamad A. Health care in gulf cooperation council countries: a review of challenges and opportunities. Cureus. (2017) 9:e1586. 10.7759/cureus.1586 29062618 PMC5650259

[20] UNDP Sustainable Development Goals by 2030. New York United Nations Development Program (2015).

[21] Chaabna K Cheema S Mamtani R. Migrants healthy worker effect and mortality trends in the Gulf Cooperation Council Countries. PLoS ONE. (2017) 12:e0179711. 10.1371/journal.pone.0179711 28632794 PMC5478152

[22] Arifeen SE Masanja H Rahman AEl. Child mortality: the challenge for India and the world. Lancet. (2017) 390:1932–3. 10.1016/S0140-6736(17)32469-8 28939095

[23] Million Death Study Collaborators Changes in cause-specific neonatal and 1-59 month child mortality in India from 2000 to 2015: a nationally representative survey. Lancet. (2017) 390:1972–80. 10.1016/S0140-6736(17)32162-1 28939096 PMC5677556

