# Supplementary material for: Child Mortality Transition in the Arabian Gulf: Wealth, Health System Reforms, and Development Goals
Source: Front Public Health. 2020 Jan 17;7:402. doi: 10.3389/fpubh.2019.00402 (PMC6978745; doi:10.3389/fpubh.2019.00402)
Supplement: Supplementary file 1 [file Table_1.docx]

**Appendix 1**

Basic yearwise data of child mortality- countrywise

| Years | Bahrain | | Kuwait | Oman | | Qatar | Saudi Arabia | UAE | Total (GCC) |
| --- | --- | --- | --- | --- | --- | --- | --- | --- | --- |
| Under 5 deaths | | | | | | | | | |
| 1961 | 1303 | | - | - | | - | - | - | - |
| 1964 | 1079 | | 1862 | - | | - | - | - | - |
| 1967 | 840 | | 2135 | 8116 | | - | - | 872 | - |
| 1974 | 446 | | 2254 | 6956 | | 247 | - | 912 | - |
| 1980 | 360 | | 1810 | 6008 | | 286 | 40065 | 984 | 49513 |
| 1990 | 329 | | 833 | 2744 | | 218 | 25494 | 792 | 30410 |
| 2000 | 182 | | 557 | 942 | | 144 | 12211 | 583 | 14619 |
| 2010 | 166 | | 624 | 781 | | 171 | 9447 | 748 | 11937 |
| 2016 | 163 | | 542 | 862 | | 213 | 8064 | 701 | 10545 |
| Number of children < 5 years | | | | | | | | | |
| 1980 | 11111 | | 50000 | 53739 | | 7901 | 408410 | 27797 | 558957 |
| 1990 | 14304 | | 47330 | 70000 | | 10481 | 570336 | 47711 | 760161 |
| 2000 | 14560 | | 43858 | 56747 | | 11520 | 552534 | 52054 | 731273 |
| 2010 | 19302 | | 57778 | 66752 | | 19000 | 601720 | 86977 | 851529 |
| 2016 | 21447 | | 64524 | 80561 | | 25059 | 625116 | 91039 | 907746 |
| Infant deaths | | | | | | | | | |
| 1957 | | 1084 | - | | - | - | - | - | - |
| 1960 | | 954 | 1128 | | - | - | - | - | - |
| 1963 | | 815 | 1353 | | 6034 | - | - | 625 | - |
| 1970 | | 467 | 1838 | | 5124 | 194 | - | 662 | - |
| 1973 | | 375 | 1836 | | 4917 | 198 | 31562 | 714 | 39602 |
| 1980 | | 304 | 1506 | | 4431 | 243 | 29880 | 843 | 37207 |
| 1990 | | 280 | 701 | | 2229 | 187 | 20571 | 683 | 24651 |
| 2000 | | 158 | 485 | | 804 | 125 | 10370 | 503 | 12445 |
| **2010** | | 142 | 538 | | 679 | 150 | 8145 | 645 | 10299 |
| **2016** | | 140 | 467 | | 745 | 185 | 6953 | 599 | 9089 |
| Number infants | | | | | | | | | |
| 1973 | | 7780 | 38330 | | 35891 | 4439 | 283321 | 11980 | 381741 |
| 1980 | | 11301 | 50707 | | 558061 | 8182 | 422631 | 28969 | 577596 |
| 1990 | | 14359 | 46424 | | 70094 | 10506 | 574609 | 48099 | 764090 |
| 2000 | | 14630 | 44091 | | 56224 | 11574 | 551596 | 52396 | 730510 |
| **2010** | | 19452 | 58479 | | 67900 | 19481 | 603333 | 88356 | 857000 |
| **2016** | | 21538 | 64861 | | 80978 | 25342 | 626396 | 90758 | 909874 |
| Neonatal mortality | | | | | | | | | |
| 1972 | | 244 | - | | 1906 | - | - | - | - |
| 1980 | | 222 | - | | 2001 | - | - | 462 | - |
| 1986 | | 231 | 660 | | 1560 | - | - | 451 | - |
| 1990 | | 216 | 456 | | 1186 | 119 | 12537 | 403 | 14917 |
| 2000 | | 74 | 293 | | 445 | 78 | 6588 | 309 | 7787 |
| 2010 | | 61 | 326 | | 390 | 92 | 5094 | 395 | 6358 |
| 2016 | | 67 | 286 | | 423 | 105 | 4338 | 361 | 5580 |
| Number of neonates | | | | | | | | | |
| 1990 | | 14400 | 45149 | | 70178 | 10531 | 580417 | 48554 | 769228 |
| 2000 | | 14800 | 44394 | | 55625 | 11818 | 553614 | 53276 | 733526 |
| 2010 | | 19677 | 59273 | | 69643 | 20000 | 606429 | 89773 | 864794 |
| 2016 | | 21613 | 65000 | | 81346 | 25610 | 628696 | 90250 | 912514 |

Source of data: UN Inter-agency Group for child Mortality Estimation (UN IGME), as 2017 release ([www.childmortality.org](http://www.childmortality.org))
