# Supplementary material for: Child Mortality Transition in the Arabian Gulf: Wealth, Health System Reforms, and Development Goals
Source: Front Public Health. 2020 Jan 17;7:402. doi: 10.3389/fpubh.2019.00402 (PMC6978745; doi:10.3389/fpubh.2019.00402)
Supplement: Supplementary file 2 [file Table_2.docx]

**Appendix 2**

Countrywise child mortality rates for various years

| Years | Bahrain | Kuwait | Oman | Qatar | Saudi Arabia | United Arab  Emirates | GCC Total |
| --- | --- | --- | --- | --- | --- | --- | --- |
| U5MR | | | | | | | |
| 1956 | 279.5 | - | - | - | - | - | - |
| 1959 | 216.9 | 157.3 | - | - | - | - | - |
| 1960 | 199.1 | 146.5 | - | - | - | 204.2 | - |
| 1961 | 182.3 | 136.3 | - | - | - | 193.8 | - |
| 1969 | 85.0 | 75.7 | 242 | 70.3 | - | 108.3 | - |
| 1970 | 76.8 | 70.9 | 229.1 | 65.8 | - | 98.5 | - |
| 1972 | 62.8 | 62.4 | 204.1 | 57.5 | 162.8 | 80.6 | - |
| 1980 | 32.4 | 36.2 | 111.8 | 36.2 | 98.1 | 35.4 | 88.58 |
| 1990 | 23.0 | 17.6 | 39.2 | 20.8 | 44.7 | 16.6 | 40.00 |
| 2000 | 12.5 | 12.7 | 16.6 | 12.5 | 22.1 | 11.2 | 19.99 |
| 2010 | 8.6 | 10.8 | 11.7 | 9.0 | 15.7 | 8.6 | 14.02 |
| 2016 | 7.6 | 8.4 | 10.7 | 8.5 | 12.9 | 7.7 | 11.62 |
| IMR | | | | | | | |
| 1956 | 186.6 | - | - | - | - | - | - |
| 1959 | 145.0 | 108.1 | - | - | - | - | - |
| 1962 | 113.7 | 88.8 | 227.8 | - | - | 123.9 | - |
| 1970 | 57.2 | 53.5 | 152.8 | 50.1 | - | 71.0 | - |
| 1972 | 48.2 | 47.9 | 137.0 | 44.6 | 111.4 | 59.6 | 103.74 |
| 1980 | 26.9 | 29.7 | 79.4 | 29.7 | 70.7 | 29.1 | 64.42 |
| 1990 | 19.5 | 15.1 | 31.8 | 17.8 | 35.8 | 14.2 | 32.26 |
| 2000 | 10.8 | 11.0 | 14.3 | 10.8 | 18.8 | 9.6 | 17.04 |
| 2010 | 7.3 | 9.2 | 10.0 | 7.7 | 13.5 | 7.3 | 12.02 |
| 2016 | 6.5 | 7.2 | 9.2 | 7.3 | 11.1 | 6.6 | 9.99 |
| NMR | | | | | | | |
| 1972 | 31.0 | - | 52.2 | - | - | - | 48.44 |
| 1980 | 19.1 | - | 34.6 | - | - | 15.2 | 26.89 |
| 1990 | 15.0 | 10.1 | 16.9 | 11.3 | 21.6 | 8.3 | 19.39 |
| 2000 | 5.0 | 6.6 | 8.0 | 6.6 | 11.9 | 5.8 | 10.62 |
| 2010 | 3.1 | 5.5 | 5.6 | 4.6 | 8.4 | 4.4 | 7.35 |
| 2016 | 3.1 | 4.4 | 5.2 | 4.1 | 6.9 | 4.0 | 6.11 |

Source of data: UN Inter-agency Group for child Mortality Estimation (UN IGME), as 2017 release ([www.childmortality.org](http://www.childmortality.org))
